# Driver genes as predictive indicators of brain metastasis in patients with advanced NSCLC: EGFR, ALK, and RET gene mutations

**DOI:** 10.1002/cam4.2706

**Published:** 2019-11-25

**Authors:** Huijuan Wang, Ziqi Wang, Guowei Zhang, Mina Zhang, Xiaojuan Zhang, Haixia Li, Xuanxuan Zheng, Zhiyong Ma

**Affiliations:** ^1^ Department of Medical Oncology The Affiliated Cancer Hospital of Zhengzhou University Henan Cancer Hospital Zhengzhou China

**Keywords:** brain metastases, clinicopathological features, driver genes, lung cancer, non‐small cell lung cancer (NSCLC), targeted therapy

## Abstract

**Background:**

A retrospective analysis verified the role of gene mutations in brain metastasis in patients with non‐small cell lung cancer (NSCLC).

**Methods:**

Data from 552 patients with advanced NSCLC treated from January 2015 to June 2017 in the Affiliated Cancer Hospital of Zhengzhou University were retrospectively analyzed. Next‐generation sequencing was used to detect mutations in eight reported driver genes and various risk factors were evaluated.

**Results:**

Of the 552 patients with advanced NSCLC, 153 (27.7%) had brain metastases. The univariate analysis showed that age (*P* = .008), gender (*P* = .016), smoking history (*P* = .010), lymph node metastasis (*P* = .003), and three driver genes, positive epidermal growth factor receptor (EGFR) mutation (*P* = .001), positive anaplastic lymphoma kinase (ALK) gene fusion (*P* = .021), and positive rearranged during transfection (RET) gene fusion (*P* = .003), were the factors influencing the incidence of brain metastasis. Logistic multivariate regression analysis revealed that positive EGFR mutation (*P* = .012), positive ALK gene fusion (*P* = .015), positive RET gene fusion (*P* = .003), pathological type (*P* = .009), lymph node N2‐3 metastasis (*P* < .001), and a younger age (*P* < .001) were independent risk factors for brain metastasis. In addition, a receiver operating characteristic (ROC) curve was plotted with the above factors with an area under the curve = 0.705 (*P < *.001).

**Conclusions:**

An EGFR mutation, ALK gene fusion, and RET gene fusion in advanced NSCLC patients play roles in brain metastasis as positive driver genes.

**Impact:**

An EGFR mutation, and ALK and RET gene fusions are risk factors for brain metastasis in advanced NSCLC patients.

## Graphical abstract

The incidence of brain metastasis in non‐small cell lung cancer (NSCLC) patients is 30%‐40%. Brain metastasis (BM) in NSCLC correlated with age, pathological type and lymph node N2‐3 metastasis. Epidermal growth factor receptor, anaplastic lymphoma kinase, and rearranged during transfection (RET) mutations were associated with BM in NSCLC patients.

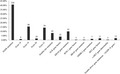

## 1 INTRODUCTION

Brain metastasis is a major cause of disease progression and death in lung cancer patients, and is also one of the most common metastatic sites of lung cancer. Non‐small cell lung cancer (NSCLC) accounts for about 80% of all lung cancer patients,^1^ and in recent years, adenocarcinoma has become the main subtype of NSCLC.^2^ Although diagnostic techniques are increasingly mature and the concept of early diagnosis and early treatment is being promoted vigorously, as the early symptoms of lung cancer are not obvious, about 70% of lung cancer patients are already at an advanced stage when they are diagnosed, and 10%‐20% of them have brain metastasis. Although the incidence of brain metastasis in NSCLC patients is slightly lower than that in SCLC patients, it can be as high as 30%‐40%.^3^ The mechanism(s) of brain metastasis and the treatment schemes are still suboptimal.

The Iressa Pan‐Asia Study (IPASS)^4^ initiated the era of targeted therapy for lung cancer, and established the role of epidermal growth factor receptor tyrosine kinase inhibitors (EGFR TKI) in EGFR mutant–positive NSCLC patients, thus opening a new direction for lung cancer research. Many researchers have begun to focus on the expression of molecular biomarkers in lung cancer, in addition to the classification of patients based on pathological types. For example, anaplastic lymphoma kinase (ALK) fusion was identified as a unique subtype of NSCLC only in 2007.^5^ From 2007 to 2011, it took only 4 years for the specific targeted drug crizotinib to be approved as a cancer treatment.^6^ In addition, the roles and mechanisms of various lung cancer–related target genes (Kirsten ratsarcoma viral oncogene homolog [KRAS], c‐ros oncogene 1, receptor tyrosine kinase [ROS‐1], BRAF, rearranged during transfection [RET], c‐MET) in the development of lung cancer have become topics of intensive study by many research groups.^7^ Therefore, we believe that among these mutant genes, there must be a driving factor for brain metastasis of lung cancer tumors. In this study, we enrolled 552 Chinese patients with advanced NSCLC and collected their clinical data. The next‐generation sequencing (NGS) method was used to detect eight genes (EGFR, ALK, KRAS, ROS‐1, BRAF, ERBB2, RET, and c‐MET) and statistical analysis was used to determine which genes were the driving factors for brain metastasis associated with lung cancer.

## 2 PATIENTS AND METHODS

### 2.1 Patients

The clinicopathological data of 552 patients who received driver genes detection for lung cancer in the Affiliated Cancer Hospital of Zhengzhou University from January 2015 to June 2017 were collected; NGS was used for gene detection. The genes for detection were EGFR, ALK, KRAS, ROS‐1, BRAF, ERBB2, RET, and c‐MET, which are eight lung cancer driver genes recommended in the NCCN guidelines. All the tests were carried out in the Center of Molecular Pathology in the Affiliated Tumor Hospital of Zhengzhou University.

### 2.2 Inclusion criteria

1. NSCLC patients with brain metastases were confirmed by magnetic resonance imaging (MRI) or positron emission tomography‐computed tomography (PET‐CT).
2. The eight genes were detected and the detection report was available.
3. The pathological type was confirmed by at least two pathologists.
4. A complete medical record was available.

### 2.3 Follow‐up method

The patients were followed‐up in a clinic or by telephone contact. The final follow‐up date was 1 February 2018 and the median follow‐up time was 41 months (21‐156 months).

### 2.4 NGS measurement

Tissue samples were obtained from patients and prepared according to the following steps:

First, the DNA sample was dissolved in 50 µL ddH_2_O/EB and DNA purification determined using Nano 2000 requiring an OD_260/280_ > 1.8 and concentration measurements using Qubit (range 30‐200 ng). The DNA quality (degradation level) was assessed using agarose gel electrophoresis.

Then, for ultrasonic crushing of target fragments (200‐300 bp), the circulation time was 40 seconds on and 60 seconds off. The number of cycles was judged according to the DNA quality, which was generally 8‐18. Terminal repair, 3 'end plus A after joint connection and product purification was established, and pre‐library amplification and product purification carried out. The concentration and fragment sizes of the purified pre‐library were readily detected. The main peak of the qualified FFPE DNA pre‐library was 250‐350 bp. Then a hybridization reaction, capture and elution, final library preparation, and purification and detection of the purified library concentration and fragment sizes were carried out.

#### 2.4.1 The analysis of detection results

The main peak of qualified FFPE DNA library was between 300 and 500 bp. The last step was sequencing.

### 2.5 Statistical methods

Statistical analysis was performed using SPSS ver. 17.0 software. A chi‐squared test was used to compare the rates when the total sample size was >40 and the theoretical frequency was ≥1. Otherwise, the Fisher's exact analysis method was used for comparisons. A chi‐squared test, Fisher exact test, or a logistic regression model was employed to screen the high risk factors for brain metastases. The predictive value of high risk factors for brain metastasis was verified by plotting a receiver operating characteristic curve (ROC), and results were statistically significant when the area under the curve (AUC) was > 0.5. Single factor survival analysis was performed by the Kaplan‐Meier method and a log‐rank test was performed (*α* = .05). Cox's and logistic regression models were used for multivariate regression analysis. A value of *P* < .05 was considered to be statistically significant.

## 3 RESULTS

### 3.1 Clinicopathological characteristic of 552 lung cancer patients

A total of 552 lung cancer patients were enrolled in the study, of whom 153 had brain metastases (27.7%) confirmed by MRI or PET‐CT. Among them, a total of 108 patients (19.6%) and 45 cases developed brain metastases metastasis at first progression diagnosed during follow‐ups. To diagnose lung cancer, 353 (63.9%) patients were treated with percutaneous lung puncture, 89 (16.1%) with bronchoscopy, 56 (10.1%) with postoperative diagnosis, 20 (3.6%) with pleural effusion, 28 (5.1%) with suspicious lymph node puncture, and 6 (1.1%) with ultrasound‐guided bronchoscopy.

The majority of the 552 patients were male (316, 57.2%), and the median age was 60 years (26‐84 years). Many patients had smoked for more than 6 months during their lifetime (342, 62.0%). At the time of diagnosis, 300 of the primary sites were in the right lung, accounting for 54.3% of cases. According to the World Health Organization classification criteria for lung cancer, adenocarcinoma is the most common pathological type, which was also the case in this study (501, 90.8%), followed by squamous cell carcinoma (20, 3.6%; Table 1).

**Table 1:** Clinicopathological information from 552 lung cancer patients

Variables (v)	Number (%)
Age (y)	
≤60	285 (51.6%)
>60	267 (48.4%)
Gender	
Male	316 (57.2%)
Female	236 (42.8%)
History of smoking	
Yes	342 (62.0%)
No	210 (38.0%)
Pathological type	
Adenocarcinoma	501 (90.8%)
Squamous cell carcinoma	20 (3.6%)
Other	31 (5.6%)
Diagnostic methods	
Percutaneous lung puncture	353 (63.9%)
Bronchoscopy	89 (16.1%)
After operation	56 (10.1%)
Pleural effusion	20 (3.6%)
Suspicious lymph node puncture	28 (5.1%)
Ultrasound guided bronchoscopy	6 (1.1%)
Diagnosis or not	
Is there brain metastasis?	
Yes	153 (27.7%)
At first visit	108 (19.6%)
During follow‐up	45 (8.2%)
No	399 (72.3%)

#### 3.1.1 Detection of eight driver genes in 552 patients with lung cancer

In all 552 patients enrolled in the study, we detected expression of eight genes (EGFR, ALK, KRAS, ROS‐1, BRAF, ERBB2, RET, and c‐MET8) by NGS. A total of 332 patients (60.1%) with a single driver gene mutation/amplification were found. EGFR mutations were most common in 226 cases (40.9%). There were 22 cases (4.0%) of ALK gene fusion positive, 55 cases (10.0%) of KRAS mutation, 3 cases (0.5%) of ROS‐1 gene fusion positive, 4 cases (0.5%) of BRAF gene mutation, 11 cases (2.0%) of RET gene fusion, 7 cases (1.3%) of ERBB2 mutation, and 3 cases (0.5%) of c‐MET gene abnormality. Therefore, the EGFR gene mutation caused problems in 40.9% of 552 patients with advanced NSCLC and EGFR could be considered as a main driven gene in these lung cancer patients.

**Figure 1:**
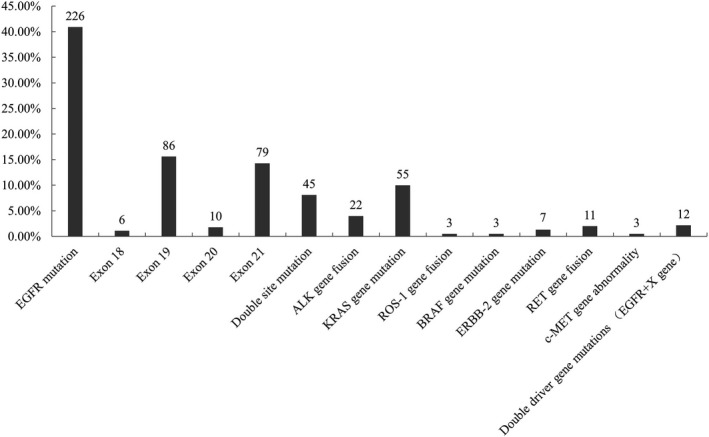
Distribution of 8 main driver gene mutations and double site gene mutations in 552 patients with lung cancer

In addition to the detection of single gene mutations, 12 patients had double‐driver gene mutations/amplifications, which are mutations/amplifications of EGFR combined with other driver genes including ALK, KRAS, BRAF, and ERBB2 mutations, and c‐MET amplification. No RET and ROS‐1 fusion gene mutation was found together with other types of driver gene mutations/amplifications. Only 3 cases of driver gene mutation or amplification co‐existed (Table S1; Figure 1). X genes in Figure 1 included the mutations of ALK, KRAS, BRAF, ERBB2, and c‐MET amplifications.

Single‐site mutation of EGFR accounted for 80.1% of patients, a total of 181 cases. There were 86 exon 19 deletion mutations (38.1%) and 76 exon 21 L858R mutations (33.6%), respectively. The rest included 3 exon 21 L861Q mutations (1.3%), 6 exon 18 mutations (2.7%), and 10 exon 20 insertion mutations (4.4%). The mutation of exon 18 was mainly a G719X missense mutation (4, 1.8%). Another 45 cases involved complex mutations, of which an exon 20 T790M mutation plus an exon 21 L858R mutations (18, 8.0%) was the most common, followed by an exon 20 T790M mutation plus an exon 19 deletion mutations (17, 7.5%). Notably, all EGFR exon 20 T790M mutations (35 cases) were in the form of co‐mutations with other sites (Table 2). Of these, 30 (13.3%) were acquired drug‐resistant mutations identified by eight‐gene detection after the progress of EGFR TKI treatment, and 5 cases (2.2%) were de novo T790M mutations.

**Table 2:** Epidermal growth factor receptor (EGFR) subtypes and their proportions

EGFR subtypes	The number of mutations	% (EGFRm+)	% (Total number)
Single‐site mutation			
Exon 18	6	2.7	1.1
Non‐frameshift deletion mutation	2	0.9	0.4
G719X missense mutation	4	1.8	0.7
Exon 19	86	38.1	15.6
Non‐frameshift deletion mutation	86	38.1	15.6
Exon 20	10	4.4	1.8
Non‐frameshift insertion mutation	10	4.4	1.8
Exon 21	79	35.0	14.3
L858R	76	33.6	13.8
L861Q	3	1.3	0.5
Double‐site mutation			
18/20	2	0.9	0.4
G719X/S768I	1	0.4	0.2
G719X/A767V	1	0.4	0.2
18/21	1	0.4	0.2
G719X/L861Q	1	0.4	0.2
19/20	18	8.0	3.3
del/T790M	17	7.5	3.1
del/R776H	1	0.4	0.2
21/20	20	8.8	3.6
L858R/T790M	18	8.0	3.3
L858R/R776H	1	0.4	0.2
L858R/A767V	1	0.4	0.2
18/18	1	0.4	0.2
G719X/E709A	1	0.4	0.2
19/19	1	0.4	0.2
del/c.2248G>C	1	0.4	0.2
21/21	2	0.9	0.4
L833V/H835L	1	0.4	0.2
L858R/L833V	1	0.4	0.2
Total	226	100	40.9

#### 3.1.2 Mutation information of 8 driver genes in patients with brain metastases

By the end of the follow‐up, a total of 153 (27.7%) patients with NSCLC had brain metastasis. There were 108 cases of brain metastases at first diagnosis, including 55 cases of EGFR mutation and most of them were from exon 19 (18.5%), followed by exon 21 (16.7%).

Forty‐five cases showed metastasis during follow‐up, of which 22 EGFR gene mutations accounted for 48.9% of them.

Next, we analyzed the effect of gene mutations on the incidence of brain metastases. Compared with patients without driver gene mutations, patients with positive EGFR mutations (*P* = .001), positive ALK gene fusion (*P* = .021), and positive RET gene fusion (*P* = .003) were more prone to brain metastasis.

The brain metastasis probability of patients with a positive KRAS mutation appeared to be higher than that of patients without driver gene mutations, but the difference was not statistically significant (*P* = .072, Table 3; Table S2).

**Table 3:** Correlation between NSCLC patients with brain metastases and different driver genes

	Brain metastases (n = 153)			Non‐ brain metastasis (n = 399)
	At first visit 108 (n, %)	Follow‐up 45 (n, %)	Total 153 (n, %)	n, %
EGFR mutation	55 (50.9)	22 (48.9)	77 (50.3)	149 (37.3)*
18 exons	2 (1.9)	0 (0)	2 (2.5)	4 (1.0)
19 exons	20 (18.5)	10 (22.2)	30 (39.0)	56 (14.0)
20 exons	4 (3.7)	1 (2.2)	5 (6.5)	5 (1.3)
21 exons	18 (16.7)	10 (22.2)	28 (3.6)	51 (12.8)
Double point mutations	11 (10.2)	1 (2.2)	12 (1.5.6)	33 (8.3)
ALK fusion mutation	4 (3.7)	5 (11.1)	9 (5.9)	13 (3.3)*
KRAS mutation	6 (5.6)	6 (13.3)	12 (7.8)	43 (10.8)*
ROS‐1 fusion mutation	0 (0)	0 (0)	0 (0.0)	3 (0.8)
BRAF mutation	0 (0)	2 (4.4)	2 (1.3)	1 (0.3)
ERBB2 mutation	0 (0)	1 (2.2)	1 (0.7)	6 (1.5)
RET fusion mutation	7 (6.5)	0 (0)	7 (4.6)	4 (1.0)
c‐MET mutation/amplification	0 (0)	1 (2.2)	1 (1.9)	2 (0.5)
Double gene mutation	2 (1.9)	2 (4.4)	4 (2.6)	8 (2.0)
No driven gene mutation	34 (31.5)	6 (13.3)	40 (26.1)	170 (42.6)

Abbreviations: ALK, anaplastic lymphoma kinase; EGFR, epidermal growth factor receptor; NSCLC, non‐small‐cell lung cancer.

Non‐brain metastasis vs brain metastases, *P < .05.

#### 3.1.3 Univariate and multivariate analyses of risk factors for NSCLC brain metastases

The univariate analysis shown in Table 4 revealed that age at onset (*P* = .008), sex (*P* = .016), smoking history (*P* = .010), gene mutation status (*P* = .001), lymph node metastasis (*P* = .003) all had an effect on the incidence of brain metastasis, but no significant difference was found among the different mutation sites of EGFR (Table 4).

**Table 4:** Univariate and multivariate analysis of the risk factors for brain metastases

Univariate analysis						Multivariate analysis			
Variables	Total	BM	%	Chi‐squared value	*P*‐value	Regression coefficient	SEM	Test statistic	*P*‐value
Age (y)									
≤60	285	93	32.6%	7.102	.008	0.025	0.006	15.188	<.001
>60	267	60	22.5%						
Gender									
Male	316	75	23.7%	5.853	.016				
Female	236	78	33.1%						
History of smoking									
Yes	210	45	21.4%	6.691	.010				
No	342	108	31.6%						
Pathological type									
Adenocarcinoma	501	145	28.9%	—	.038	—	—	9.366	.009
Squamous cell carcinoma	20	1	5.0%			−3.678	0.411	2.848	.038
Others	31	7	22.6%			0.641	0.276	5.405	.091
Primary site									
Left lung	245	71	29.0%	—	.705				
Right lung	300	81	27.0%						
Mediastinum	4	0	0%						
Double lung	3	1	33.3%						
Driver gene mutation									
EGFR	226	77	34.1%	—	.001	0.456	0.180	6.384	.012
ALK	22	9	40.9%			2.182	0.901	5.870	.015
KRAS	55	12	21.8%						
ROS‐1	3	0	0%						
BRAF	3	2	66.7%						
RET	11	7	63.6%			1.361	0.465	8.557	.003
ERBB2	7	1	14.3%						
C‐MET	3	1	33.3%						
Double gene mutation	12	4	33.3%						
No	210	40	19.0%						
EGFR									
21 exon	79	28	35.4%	0.324	.851				
19 exon	86	30	34.9%						
Others	61	19	31.1%						
Lymph node metastasis									
N0‐1	255	59	23.3%	8.924	.003	−1.031	0.228	20.461	<.001
N2‐3	297	104	35.1%						
Imaging features of primary lesions									
Central type	202	63	31.2%	—	.366				
Peripheral type	347	90	25.9%						
Dispersion type	3	1	33.3%						
Constant						−6.449	2.322	7.713	.005

Finally, all factors were included in logistic multivariate analysis and it was found that only a positive EGFR mutation (*P* = .012), a positive ALK gene fusion (*P* = .015), a positive RET gene fusion (*P* = .003), pathological type (*P* = .009), lymph node N2‐3 metastasis (*P* < .001), and <60 years old (*P* < .001) were independent risk factors for brain metastasis (Table 4).

#### 3.1.4 Driving genes as predictors for a high risk of developing brain metastases

**Figure 2:**
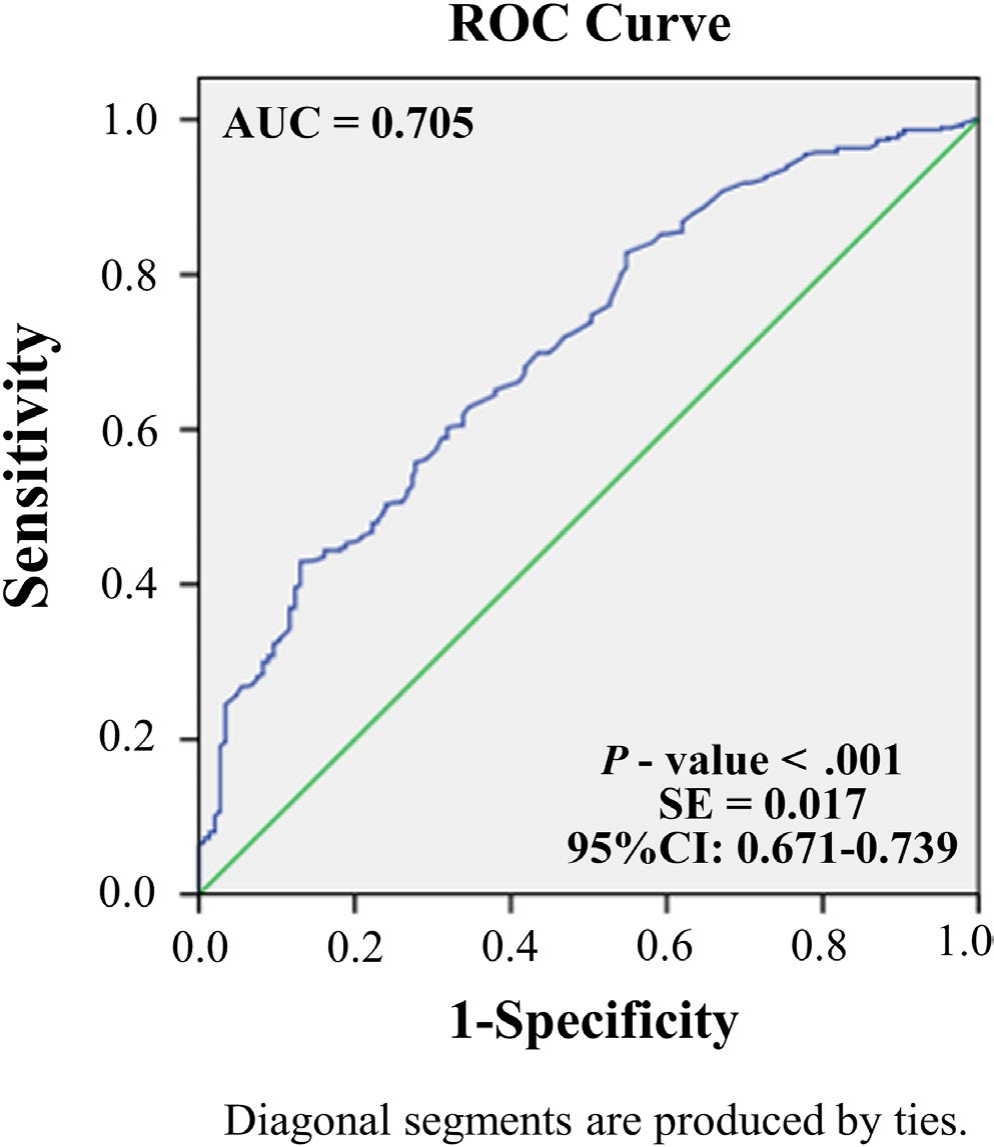
Epidermal growth factor receptor (EGFR), RET, anaplastic lymphoma kinase (ALK) gene status, lymph node metastasis, age, and pathological type including multifactor receiver operating characteristic (ROC) curve

In order to establish the value of logistic multivariate regression analysis for identifying risk factors for brain metastasis, ROC curves were plotted by using the factors with statistical significance in multi‐factor analysis, namely EGFR, RET, ALK gene status, lymph node metastasis, age, and pathological type. The results revealed an AUC = 0.705 (*P* < .001, SE = 0.017, 95% CI: 0.671‐0.739; Figure 2).

## 4 DISCUSSION

This study evaluated the factors which might correlate with brain metastases at initial diagnosis or during first progression of NSCLC and is the first large‐scale study of the effects of combined detection of NSCLC multi‐driver genes on the incidence of brain metastasis. It was found that only positive EGFR mutations (*P* = .012), positive ALK gene fusion (*P* = .015), and positive RET gene fusion (*P* = .003) were independent risk factors for brain metastasis, and that a young age, lymph node metastasis, and histological type (adenocarcinoma) were also independent risk factors for brain metastasis. This study is the first to report that the positive RET fusion gene has a significant effect on the occurrence of brain metastasis. Although there are few cases of partial mutation, this finding is still of great clinical value.

It has been shown that 10%‐20% of patients with advanced NSCLC developed brain metastasis on first diagnosis and that 30%‐40% of patients had brain metastasis during the progression of the disease.^3^ In this study, 19.6% of the initial patients and 27.7% of the follow‐up patients had brain metastases, findings that were basically consistent with previous reports. The mechanisms underlying brain metastasis in lung cancer patients remain unclear. Recent studies have found that integrin, E‐cadherin, vascular endothelial growth factor receptor (V‐EGFR), caspase‐3, Wnt/TCF, PI3K‐Akt pathway, endothelin, and endothelin receptor pathways play important roles in the development of brain metastases.^8^, ^9^ Although the pathogenesis of brain metastasis is still not fully understood, there can be no doubt that the occurrence of brain metastasis is the result of multiple factors and pathways. However, the relationship between the RET fusion gene and brain metastasis can be referenced by the mechanism of RET gene and tumor metastasis.

Patients with brain metastasis and lung cancer have a very poor prognosis, and the survival time without treatment is typically only 1‐2 months.^10^ Even patients who have received whole‐brain radiotherapy have a survival time of only 4‐6 months.^11^ Therefore, screening and prevention of high‐risk brain metastases are very important endeavors. The discovery of high risk factors in clinical practice will undoubtedly point to some new directions for basic research on the mechanisms driving brain metastases. Previous studies have shown that the risk factors for brain metastasis in lung cancer patients mainly include a younger age at onset, pathological type of non‐squamous cell carcinoma, and the late N stage.^12^ In the present study, the incidence of brain metastasis was higher in patients with an onset age ≤60 years (*P* = .012), lymph node N2‐N3 metastasis (*P* = .000), and adenocarcinoma (*P* = .009), which are consistent with previously reported findings. Furthermore, there was a significant correlation between EGFR mutation positive (*P* = .001), ALK gene fusion positive (*P* = .021), RET gene fusion positive (*P* = .003), and brain metastasis.

In recent years, there have been a number of published research reports about the effect of driver gene mutations on brain metastasis in lung cancer patients, among which EGFR is the most concerning factor. In a study of an Eastern population by Matsumoto et al,^13^ it was shown that 63% of patients with brain metastasis had an EGFR mutation, and the study of Huang et al^14^ reported that the number was 30% in a Taiwanese population, while it was 40% and 50% in the studies of Gow et al^15^ and Wu et al,^16^ respectively. In our study, the EGFR mutation rate of patients with brain metastasis was 50.3%, which was consistent with the results of previous studies but higher than the EGFR mutation rate (about 30%) reported in the previous overall population. Subsequent multifactor analysis showed that EGFR was indeed an independent risk factor for brain metastasis in NSCLC patients (*P* = .012), a finding consistent with the results of studies by Eichler et al^10^ and Liu et al.^17^ It may be attributed to the fact that EGFR mutations are involved in the occurrence of brain metastasis from lung cancer, or it may be due to the use of EGFR‐TKI that leads to a longer survival in patients with an EGFR mutation. In addition, patients with an EGFR exon 19 deletion mutation were reported to be more prone to brain metastasis than patients with an exon 21 L858R mutation. However, the selected sample of patients with brain metastases at the time of initial diagnosis may not have accurately established the risk of brain metastases during the course of the disease. In our subgroup analysis, no difference was found in the incidence of brain metastases between groups with different types of EGFR mutations. The clear risk factor for brain metastasis is an EGFR mutation and the effect of different mutation subtypes on brain metastasis remains to be further studied.

Anaplastic lymphoma kinase gene fusion is another driver gene of great significance for advanced NSCLC patients in addition to EGFR mutations. ALK protein kinase inhibitors greatly improve the prognosis of patients with ALK‐positive NSCLC,^18^ and it has been noted in the clinic that patients with seemingly positive ALK fusion genes are more likely to develop brain metastases. In the Profile 1007 study, 35% of ALK fusion gene positive patients had brain metastases at the screening stage.^19^ In this study, it was found that over 40% of patients with positive ALK fusion protein presented with brain metastasis, which was much higher than those with negative driver gene tests (19.1%). It is also noteworthy that 7 out of 11 RET fusion gene‐positive patients (63.6%) had brain metastases during follow‐up. Multivariate analysis later confirmed that a positive RET fusion protein gene was an independent risk factor for brain metastasis in lung cancer patients (*P* = .003). This is the first report about the relationship between the RET fusion gene and brain metastasis, a topic which deserves much further attention. The findings remind us that the RET fusion gene is likely to play an important role in the occurrence of brain metastasis in lung cancer patients, and it is expected that further basic research will be conducted to elucidate the mechanisms underlying this clinical phenomenon. There was no relationship between KRAS, BRAF, ROS‐1, ERBB2, c‐MET, double gene mutation, and brain metastasis.

Therefore, as the role of more driver genes and their prognostic value are clarified, targeted therapy will play an increasingly important role in the treatment of advanced NSCLC. Currently, the driver genes recommended in NCCN guidelines for advanced NSCLC detection include EGFR, ALK, KRAS, RET, ROS‐1, ERBB2, BRAF, and c‐MET, among which patients with EGFR, ALK, and ROS1 positive^4^, ^6^, ^20^ are recommended to use first‐line corresponding targeted drug therapy. Compared with Sanger sequencing, NGS is characterized by high throughput, high sensitivity and a fast sequencing speed.^21^ It is the most stable and widely used sequencing technology available today. All the enrolled patients in this study were tested for eight driver genes, EGFR, ALK, KRAS, RET, ROS‐1, ERBB2, BRAF, and c‐MET by NGS.

This comprehensive analysis of the driver genes has fewer confounding factors than previous studies of single gene mutations such as EGFR or ALK, and can more accurately assess the effects of various gene mutations on the incidence of brain metastasis. The ROC curve can better describe the impact of two categorical variables on the outcome. In order to verify the predictive value of the above risk factors for brain metastasis, we included them in the ROC curve, showing that the AUC = 0.705 and *P* < .001, indicating that a comprehensive assessment of the above risk factors and gene mutations will be of great value in predicting brain metastasis.

## 5 CONCLUSIONS

In patients with advanced NSCLC, EGFR mutations, ALK gene fusion, RET gene fusion, younger age (≤60 years), lymph node N2‐N3 metastasis and the pathological type of adenocarcinoma are all independent risk factors for brain metastasis. Combined detection of EGFR mutations, ALK gene fusion, and RET gene fusion for the eight major driving genes may be a predictive factor for brain metastasis in NSCLC patients. Its exact clinical threshold needs further validation in RCT studies.

## AUTHORS' CONTRIBUTIONS

HW, MZ, and ZM were responsible for the conception and design of the study. HW, ZW, GZ, MZ, XZ, HL, XZ, and ZM were responsible for acquisition and analysis of data; furthermore, HW, ZW, GZ, and MZ were in charge of statistical analysis. HW, ZW, HL, and XZ drafted the manuscript; HW and ZM revised and commented on the draft, and HW, ZW, and ZM approved the final version of the manuscript.

## DECLARATIONS

### ETHICS APPROVAL AND CONSENT TO PARTICIPATE

The ethical committee of the Henan Cancer Hospital approved this study and written informed consent was obtained from all patients.

### CONSENT FOR PUBLICATION

Consent for publication was obtained from all patients.

### CONFLICT OF INTEREST

The authors declare there are no potential conflicts of interest.

## Data Availability

All data generated or analyzed during this study are included in this published article.
