# Supplementary material for: Driver genes as predictive indicators of brain metastasis in patients with advanced NSCLC: EGFR, ALK, and RET gene mutations
Source: Cancer Med. 2019 Nov 25;9(2):487–95. doi: 10.1002/cam4.2706 (PMC6970058; doi:10.1002/cam4.2706)
Supplement: Supplementary file 1 [file CAM4-9-487-s001.docx]

**Supplementary Table 1.** Driver gene mutations in 552 patients with lung cancer

| Driver gene | N | % (positive / overall patients) |
| --- | --- | --- |
| EGFR mutation | 226 | 40.9% |
| Exon 18 | 6 | 1.1% |
| Exon 19 | 86 | 15.6% |
| Exon 20 | 10 | 1.8% |
| Exon 21 | 79 | 14.3% |
| Double site mutation | 45 | 8.1% |
| ALK gene fusion | 22 | 4.0% |
| KRAS gene mutation | 55 | 10.0% |
| ROS-1 gene fusion | 3 | 0.5% |
| BRAF gene mutation | 3 | 0.5% |
| ERBB-2 gene mutation | 7 | 1.3% |
| RET gene fusion | 11 | 2.0% |
| c-MET gene abnormality | 3 | 0.5% |
| Double driver gene mutations  (EGFR + X gene) | 12 | 2.2% |
| Total | 552 | 100% |

**Supplementary Table 2. Mutation type of the driving gene related to the**

**incidence of brain metastases**

| Mutations | NSCLC Total | Brain metastases BM153 | Chi-squared value | *P-*value |
| --- | --- | --- | --- | --- |
| Driver gene mutation |  |  |  |  |
| Yes | 343 | 112 | 11.015 | 0.001 |
| EGFR | 226 | 77 | 11.475 | 0.001 |
| ALK | 22 | 9 | 5.321 | 0.021 |
| RET | 11 | 7 | - | 0.003 |
| KRAS | 55 | 12 | 3.239 | 0.072 |
| ROS-1 | 3 | 0 | - | 1.000 |
| BRAF | 3 | 2 | - | 0.105 |
| ERBB2 | 7 | 1 | - | 1.000 |
| c-MET | 3 | 1 | - | 0.484 |
| Double gene mutation | 12 | 4 | - |  |
| No | 210 | 40 | - |  |
| Total | 552 | 153 | - |  |
